# Supplementary material for: Mutual Influence of Human Cytochrome P450 Enzymes and UDP-Glucuronosyltransferases on Their Respective Activities in Recombinant Fission Yeast
Source: Biomedicines. 2023 Jan 19;11(2):281. doi: 10.3390/biomedicines11020281 (PMC9953201; doi:10.3390/biomedicines11020281)
Supplement: Supplementary file 1 [file biomedicines-11-00281-s001.zip › biomedicines-2114044-supplementary.pdf]

Supplemental Information

Table S1. Strains used in this study.

Serial number	Strain name	Parental strain(s)	Genotype	Expressed protein(s)	Reference
**Haploids**					
1	JMN11	None	h+ ade6-M210 ura4-D18 his3.Δ1	None	This study
2	JMN12	None	h- ade6-M216 ura4-D18 his3.Δ1	None	This study
3	SAN1	JMN11	h+ ade6-M210 ura4-D18 his3.Δ1 leu1::pCAD1	None	This study
4	SAN2	JMN11	h+ ade6-M210 ura4-D18 his3.Δ1 leu1::pCAD1-CPR	CPR	This study
5	SAN3	JMN12	h- ade6-M216 ura4-D18 his3.Δ1 leu1::pCAD1	None	This study
6	SAN4	JMN12	h- ade6-M216 ura4-D18 his3.Δ1 leu1::pCAD1-UGT1A1	UGT1A1	This study
7	SAN5	JMN12	h- ade6-M216 ura4-D18 his3.Δ1 leu1::pCAD1-UGT1A3	UGT1A3	This study
8	SAN6	JMN12	h- ade6-M216 ura4-D18 his3.Δ1 leu1::pCAD1-UGT1A4	UGT1A4	This study
9	SAN7	JMN12	h- ade6-M216 ura4-D18 his3.Δ1 leu1::pCAD1-UGT1A5	UGT1A5	This study
10	SAN8	JMN12	h- ade6-M216 ura4-D18 his3.Δ1 leu1::pCAD1-UGT1A6	UGT1A6	This study
11	SAN9	JMN12	h- ade6-M216 ura4-D18 his3.Δ1 leu1::pCAD1-UGT1A7	UGT1A7	This study
12	SAN10	JMN12	h- ade6-M216 ura4-D18 his3.Δ1 leu1::pCAD1-UGT1A8	UGT1A8	This study
13	SAN11	JMN12	h- ade6-M216 ura4-D18 his3.Δ1 leu1::pCAD1-UGT1A9	UGT1A9	This study
14	SAN12	JMN12	h- ade6-M216 ura4-D18 his3.Δ1 leu1::pCAD1-UGT1A10	UGT1A10	This study
15	SAN13	JMN12	h- ade6-M216 ura4-D18 his3.Δ1 leu1::pCAD1-UGT2A1	UGT2A1	This study
16	SAN14	JMN12	h- ade6-M216 ura4-D18 his3.Δ1 leu1::pCAD1-UGT2A2	UGT2A2	This study
17	SAN15	JMN12	h- ade6-M216 ura4-D18 his3.Δ1 leu1::pCAD1-UGT2A3	UGT2A3	This study
18	SAN16	JMN12	h- ade6-M216 ura4-D18 his3.Δ1 leu1::pCAD1-UGT2B4	UGT2B4	This study
19	SAN17	JMN12	h- ade6-M216 ura4-D18 his3.Δ1 leu1::pCAD1-UGT2B7	UGT2B7	This study
20	SAN18	JMN12	h- ade6-M216 ura4-D18 his3.Δ1 leu1::pCAD1-UGT2B10	UGT2B10	This study
21	SAN19	JMN12	h- ade6-M216 ura4-D18 his3.Δ1 leu1::pCAD1-UGT2B11	UGT2B11	This study
22	SAN20	JMN12	h- ade6-M216 ura4-D18 his3.Δ1 leu1::pCAD1-UGT2B15	UGT2B15	This study

23	SAN21	JMN12	h- ade6-M216 ura4-D18 his3.Δ1 leu1::pCAD1-UGT2B17	UGT2B17	This study
24	SAN22	JMN12	h- ade6-M216 ura4-D18 his3.Δ1 leu1::pCAD1-UGT2B28	UGT2B28	This study
**Diploids with CPR and UGTs**					
25	SAN100	SAN2, SAN3	h+/h- ade6-M210/ade6-M216 ura4-D18/ura4-D18 his3.Δ1/his3.Δ1 leu1::pCAD1-CPR/leu1::pCAD1	CPR	This study
26	SAN101	SAN2, SAN4	h+/h- ade6-M210/ade6-M216 ura4-D18/ura4-D18 his3.Δ1/his3.Δ1 leu1::pCAD1-CPR/leu1::pCAD1-UGT1A1	CPR, UGT1A1	This study
27	SAN102	SAN2, SAN5	h+/h- ade6-M210/ade6-M216 ura4-D18/ura4-D18 his3.Δ1/his3.Δ1 leu1::pCAD1-CPR/leu1::pCAD1-UGT1A3	CPR, UGT1A3	This study
28	SAN103	SAN2, SAN6	h+/h- ade6-M210/ade6-M216 ura4-D18/ura4-D18 his3.Δ1/his3.Δ1 leu1::pCAD1-CPR/leu1::pCAD1-UGT1A4	CPR, UGT1A4	This study
29	SAN104	SAN2, SAN7	h+/h- ade6-M210/ade6-M216 ura4-D18/ura4-D18 his3.Δ1/his3.Δ1 leu1::pCAD1-CPR/leu1::pCAD1-UGT1A5	CPR, UGT1A5	This study
30	SAN105	SAN2, SAN8	h+/h- ade6-M210/ade6-M216 ura4-D18/ura4-D18 his3.Δ1/his3.Δ1 leu1::pCAD1-CPR/leu1::pCAD1-UGT1A6	CPR, UGT1A6	This study
31	SAN106	SAN2, SAN9	h+/h- ade6-M210/ade6-M216 ura4-D18/ura4-D18 his3.Δ1/his3.Δ1 leu1::pCAD1-CPR/leu1::pCAD1-UGT1A7	CPR, UGT1A7	This study
32	SAN107	SAN2, SAN10	h+/h- ade6-M210/ade6-M216 ura4-D18/ura4-D18 his3.Δ1/his3.Δ1 leu1::pCAD1-CPR/leu1::pCAD1-UGT1A8	CPR, UGT1A8	This study
33	SAN108	SAN2, SAN11	h+/h- ade6-M210/ade6-M216 ura4-D18/ura4-D18 his3.Δ1/his3.Δ1 leu1::pCAD1-CPR/leu1::pCAD1-UGT1A9	CPR, UGT1A9	This study
34	SAN109	SAN2, SAN12	h+/h- ade6-M210/ade6-M216 ura4-D18/ura4-D18 his3.Δ1/his3.Δ1 leu1::pCAD1-CPR/leu1::pCAD1-UGT1A10	CPR, UGT1A10	This study
35	SAN110	SAN2, SAN13	h+/h- ade6-M210/ade6-M216 ura4-D18/ura4-D18 his3.Δ1/his3.Δ1 leu1::pCAD1-CPR/leu1::pCAD1-UGT2A1	CPR, UGT2A1	This study
36	SAN111	SAN2, SAN14	h+/h- ade6-M210/ade6-M216 ura4-D18/ura4-D18 his3.Δ1/his3.Δ1 leu1::pCAD1-CPR/leu1::pCAD1-UGT2A2	CPR, UGT2A2	This study
37	SAN112	SAN2, SAN15	h+/h- ade6-M210/ade6-M216 ura4-D18/ura4-D18 his3.Δ1/his3.Δ1 leu1::pCAD1-CPR/leu1::pCAD1-UGT2A3	CPR, UGT2A3	This study
38	SAN113	SAN2, SAN16	h+/h- ade6-M210/ade6-M216 ura4-D18/ura4-D18 his3.Δ1/his3.Δ1 leu1::pCAD1-CPR/leu1::pCAD1-UGT2B4	CPR, UGT2B4	This study
39	SAN114	SAN2, SAN17	h+/h- ade6-M210/ade6-M216 ura4-D18/ura4-D18 his3.Δ1/his3.Δ1 leu1::pCAD1-CPR/leu1::pCAD1-UGT2B7	CPR, UGT2B7	This study

40	SAN115	SAN2, SAN18	h+/h- ade6-M210/ade6-M216 ura4-D18/ura4-D18 his3.Δ1/his3.Δ1 leu1::pCAD1-CPR/leu1::pCAD1-UGT2B10	CPR, UGT2B10	This study
41	SAN116	SAN2, SAN19	h+/h- ade6-M210/ade6-M216 ura4-D18/ura4-D18 his3.Δ1/his3.Δ1 leu1::pCAD1-CPR/leu1::pCAD1-UGT2B11	CPR, UGT2B11	This study
42	SAN117	SAN2, SAN20	h+/h- ade6-M210/ade6-M216 ura4-D18/ura4-D18 his3.Δ1/his3.Δ1 leu1::pCAD1-CPR/leu1::pCAD1-UGT2B15	CPR, UGT2B15	This study
43	SAN118	SAN2, SAN21	h+/h- ade6-M210/ade6-M216 ura4-D18/ura4-D18 his3.Δ1/his3.Δ1 leu1::pCAD1-CPR/leu1::pCAD1-UGT2B17	CPR, UGT2B17	This study
44	SAN119	SAN2, SAN22	h+/h- ade6-M210/ade6-M216 ura4-D18/ura4-D18 his3.Δ1/his3.Δ1 leu1::pCAD1-CPR/leu1::pCAD1-UGT2B28	CPR, UGT2B28	This study
**Diploids with UGTs**					
45	SAN120	SAN1, SAN3	h+/h- ade6-M210/ade6-M216 ura4-D18/ura4-D18 his3.Δ1/his3.Δ1 leu1::pCAD1/leu1::pCAD1	None	This Study
46	SAN123	SAN2, SAN6	h+/h- ade6-M210/ade6-M216 ura4-D18/ura4-D18 his3.Δ1/his3.Δ1 leu1::pCAD1/leu1::pCAD1-UGT1A4	UGT1A4	This study
47	SAN128	SAN2, SAN11	h+/h- ade6-M210/ade6-M216 ura4-D18/ura4-D18 his3.Δ1/his3.Δ1 leu1::pCAD1/leu1::pCAD1-UGT1A9	UGT1A9	This study
48	SAN132	SAN2, SAN15	h+/h- ade6-M210/ade6-M216 ura4-D18/ura4-D18 his3.Δ1/his3.Δ1 leu1::pCAD1/leu1::pCAD1-UGT2A3	UGT2A3	This study
49	SAN134	SAN1, SAN17	h+/h- ade6-M210/ade6-M216 ura4-D18/ura4-D18 his3.Δ1/his3.Δ1 leu1::pCAD1/leu1::pCAD1-UGT2B7	UGT2B7	This study
50	SAN139	SAN2, SAN22	h+/h- ade6-M210/ade6-M216 ura4-D18/ura4-D18 his3.Δ1/his3.Δ1 leu1::pCAD1/leu1::pCAD1-UGT2B28	UGT2B28	This study
**Diploids with CYP2C9**					
51	SAN200	SAN100	h+/h- ade6-M210/ade6-M216 ura4-D18/ura4-D18 his3.Δ1/his3.Δ1 leu1::pCAD1-CPR/leu1::pCAD1/pREP1-CYP2C9	CPR, CYP2C9	This study
52	SAN201	SAN101	h+/h- ade6-M210/ade6-M216 ura4-D18/ura4-D18 his3.Δ1/his3.Δ1 leu1::pCAD1-CPR/leu1::pCAD1-UGT1A1	CPR, UGT1A1, CYP2C9	This study
53	SAN202	SAN102	h+/h- ade6-M210/ade6-M216 ura4-D18/ura4-D18 his3.Δ1/his3.Δ1 leu1::pCAD1-CPR/leu1::pCAD1-UGT1A3/pREP1-CYP2C9	CPR, UGT1A3, CYP2C9	This study
54	SAN203	SAN103	h+/h- ade6-M210/ade6-M216 ura4-D18/ura4-D18 his3.Δ1/his3.Δ1 leu1::pCAD1-CPR/leu1::pCAD1-UGT1A4/pREP1-CYP2C9	CPR, UGT1A4, CYP2C9	This study

55	SAN204	SAN104	h+/h- ade6-M210/ade6-M216 ura4-D18/ura4-D18 his3.Δ1/his3.Δ1 leu1::pCAD1-CPR/leu1::pCAD1-UGT1A5/pREP1-CYP2C9	CPR, UGT1A5, CYP2C9	This study
56	SAN205	SAN105	h+/h- ade6-M210/ade6-M216 ura4-D18/ura4-D18 his3.Δ1/his3.Δ1 leu1::pCAD1-CPR/leu1::pCAD1-UGT1A6/pREP1-CYP2C9	CPR, UGT1A6, CYP2C9	This study
57	SAN206	SAN106	h+/h- ade6-M210/ade6-M216 ura4-D18/ura4-D18 his3.Δ1/his3.Δ1 leu1::pCAD1-CPR/leu1::pCAD1-UGT1A7/pREP1-CYP2C9	CPR, UGT1A7, CYP2C9	This study
58	SAN207	SAN107	h+/h- ade6-M210/ade6-M216 ura4-D18/ura4-D18 his3.Δ1/his3.Δ1 leu1::pCAD1-CPR/leu1::pCAD1-UGT1A8/pREP1-CYP2C9	CPR, UGT1A8, CYP2C9	This study
59	SAN208	SAN108	h+/h- ade6-M210/ade6-M216 ura4-D18/ura4-D18 his3.Δ1/his3.Δ1 leu1::pCAD1-CPR/leu1::pCAD1-UGT1A9/pREP1-CYP2C9	CPR, UGT1A9, CYP2C9	This study
60	SAN209	SAN109	h+/h- ade6-M210/ade6-M216 ura4-D18/ura4-D18 his3.Δ1/his3.Δ1 leu1::pCAD1-CPR/leu1::pCAD1-UGT1A10/pREP1-CYP2C9	CPR, UGT1A10, CYP2C9	This study
61	SAN210	SAN110	h+/h- ade6-M210/ade6-M216 ura4-D18/ura4-D18 his3.Δ1/his3.Δ1 leu1::pCAD1-CPR/leu1::pCAD1-UGT2A1/pREP1-CYP2C9	CPR, UGT2A1, CYP2C9	This study
62	SAN211	SAN111	h+/h- ade6-M210/ade6-M216 ura4-D18/ura4-D18 his3.Δ1/his3.Δ1 leu1::pCAD1-CPR/leu1::pCAD1-UGT2A2/pREP1-CYP2C9	CPR, UGT2A2, CYP2C9	This study
63	SAN212	SAN112	h+/h- ade6-M210/ade6-M216 ura4-D18/ura4-D18 his3.Δ1/his3.Δ1 leu1::pCAD1-CPR/leu1::pCAD1-UGT2A3/pREP1-CYP2C9	CPR, UGT2A3, CYP2C9	This study
64	SAN213	SAN113	h+/h- ade6-M210/ade6-M216 ura4-D18/ura4-D18 his3.Δ1/his3.Δ1 leu1::pCAD1-CPR/leu1::pCAD1-UGT2B4/pREP1-CYP2C9	CPR, UGT2B4, CYP2C9	This study
65	SAN214	SAN114	h+/h- ade6-M210/ade6-M216 ura4-D18/ura4-D18 his3.Δ1/his3.Δ1 leu1::pCAD1-CPR/leu1::pCAD1-UGT2B7/pREP1-CYP2C9	CPR, UGT2B7, CYP2C9	This study
66	SAN215	SAN115	h+/h- ade6-M210/ade6-M216 ura4-D18/ura4-D18 his3.Δ1/his3.Δ1 leu1::pCAD1-CPR/leu1::pCAD1-UGT2B10/pREP1-CYP2C9	CPR, UGT2B10, CYP2C9	This study
67	SAN216	SAN116	h+/h- ade6-M210/ade6-M216 ura4-D18/ura4-D18 his3.Δ1/his3.Δ1 leu1::pCAD1-CPR/leu1::pCAD1-UGT2B11/pREP1-CYP2C9	CPR, UGT2B11, CYP2C9	This study
68	SAN217	SAN117	h+/h- ade6-M210/ade6-M216 ura4-D18/ura4-D18 his3.Δ1/his3.Δ1 leu1::pCAD1-CPR/leu1::pCAD1-UGT2B15/pREP1-CYP2C9	CPR, UGT2B15, CYP2C9	This study
69	SAN218	SAN118	h+/h- ade6-M210/ade6-M216 ura4-D18/ura4-D18 his3.Δ1/his3.Δ1 leu1::pCAD1-CPR/leu1::pCAD1-UGT2B17/pREP1-CYP2C9	CPR, UGT2B17, CYP2C9	This study
70	SAN219	SAN119	h+/h- ade6-M210/ade6-M216 ura4-D18/ura4-D18 his3.Δ1/his3.Δ1 leu1::pCAD1-CPR/leu1::pCAD1-UGT2B28/pREP1-CYP2C9	CPR, UGT2B28, CYP2C9	This study

Diploids with CYP2D6					
71	SAN300	SAN100	h+/h- ade6-M210/ade6-M216 ura4-D18/ura4-D18 his3.Δ1/his3.Δ1 leu1::pCAD1-CPR/leu1::pCAD1/pREP1-CYP2D6	CPR, CYP2D6	This study
72	SAN301	SAN101	h+/h- ade6-M210/ade6-M216 ura4-D18/ura4-D18 his3.Δ1/his3.Δ1 leu1::pCAD1-CPR/leu1::pCAD1-UGT1A1/pREP1-CYP2D6	CPR, UGT1A1, CYP2D6	This study
73	SAN302	SAN102	h+/h- ade6-M210/ade6-M216 ura4-D18/ura4-D18 his3.Δ1/his3.Δ1 leu1::pCAD1-CPR/leu1::pCAD1-UGT1A3/pREP1-CYP2D6	CPR, UGT1A3, CYP2D6	This study
74	SAN303	SAN103	h+/h- ade6-M210/ade6-M216 ura4-D18/ura4-D18 his3.Δ1/his3.Δ1 leu1::pCAD1-CPR/leu1::pCAD1-UGT1A4/pREP1-CYP2D6	CPR, UGT1A4, CYP2D6	This study
75	SAN304	SAN104	h+/h- ade6-M210/ade6-M216 ura4-D18/ura4-D18 his3.Δ1/his3.Δ1 leu1::pCAD1-CPR/leu1::pCAD1-UGT1A5/pREP1-CYP2D6	CPR, UGT1A5, CYP2D6	This study
76	SAN305	SAN105	h+/h- ade6-M210/ade6-M216 ura4-D18/ura4-D18 his3.Δ1/his3.Δ1 leu1::pCAD1-CPR/leu1::pCAD1-UGT1A6/pREP1-CYP2D6	CPR, UGT1A6, CYP2D6	This study
77	SAN306	SAN106	h+/h- ade6-M210/ade6-M216 ura4-D18/ura4-D18 his3.Δ1/his3.Δ1 leu1::pCAD1-CPR/leu1::pCAD1-UGT1A7/pREP1-CYP2D6	CPR, UGT1A7, CYP2D6	This study
78	SAN307	SAN107	h+/h- ade6-M210/ade6-M216 ura4-D18/ura4-D18 his3.Δ1/his3.Δ1 leu1::pCAD1-CPR/leu1::pCAD1-UGT1A8/pREP1-CYP2D6	CPR, UGT1A8, CYP2D6	This study
79	SAN308	SAN108	h+/h- ade6-M210/ade6-M216 ura4-D18/ura4-D18 his3.Δ1/his3.Δ1 leu1::pCAD1-CPR/leu1::pCAD1-UGT1A9/pREP1-CYP2D6	CPR, UGT1A9, CYP2D6	This study
80	SAN309	SAN109	h+/h- ade6-M210/ade6-M216 ura4-D18/ura4-D18 his3.Δ1/his3.Δ1 leu1::pCAD1-CPR/leu1::pCAD1-UGT1A10/pREP1-CYP2D6	CPR, UGT1A10, CYP2D6	This study
81	SAN310	SAN110	h+/h- ade6-M210/ade6-M216 ura4-D18/ura4-D18 his3.Δ1/his3.Δ1 leu1::pCAD1-CPR/leu1::pCAD1-UGT2A1/pREP1-CYP2D6	CPR, UGT2A1, CYP2D6	This study
82	SAN311	SAN111	h+/h- ade6-M210/ade6-M216 ura4-D18/ura4-D18 his3.Δ1/his3.Δ1 leu1::pCAD1-CPR/leu1::pCAD1-UGT2A2/pREP1-CYP2D6	CPR, UGT2A2, CYP2D6	This study
83	SAN312	SAN112	h+/h- ade6-M210/ade6-M216 ura4-D18/ura4-D18 his3.Δ1/his3.Δ1 leu1::pCAD1-CPR/leu1::pCAD1-UGT2A3/pREP1-CYP2D6	CPR, UGT2A3, CYP2D6	This study
84	SAN313	SAN113	h+/h- ade6-M210/ade6-M216 ura4-D18/ura4-D18 his3.Δ1/his3.Δ1 leu1::pCAD1-CPR/leu1::pCAD1-UGT2B4/pREP1-CYP2D6	CPR, UGT2B4, CYP2D6	This study
85	SAN314	SAN114	h+/h- ade6-M210/ade6-M216 ura4-D18/ura4-D18 his3.Δ1/his3.Δ1 leu1::pCAD1-CPR/leu1::pCAD1-UGT2B7/pREP1-CYP2D6	CPR, UGT2B7, CYP2D6	This study
86	SAN315	SAN115	h+/h- ade6-M210/ade6-M216 ura4-D18/ura4-D18 his3.Δ1/his3.Δ1 leu1::pCAD1-CPR/leu1::pCAD1-UGT2B10/pREP1-CYP2D6	CPR, UGT2B10, CYP2D6	This study

87	SAN316	SAN116	h+/h- ade6-M210/ade6-M216 ura4-D18/ura4-D18 his3.Δ1/his3.Δ1 leu1::pCAD1-CPR/leu1::pCAD1-UGT2B11/pREP1-CYP2D6	CPR, UGT2B11, CYP2D6	This study
88	SAN317	SAN117	h+/h- ade6-M210/ade6-M216 ura4-D18/ura4-D18 his3.Δ1/his3.Δ1 leu1::pCAD1-CPR/leu1::pCAD1-UGT2B15/pREP1-CYP2D6	CPR, UGT2B15, CYP2D6	This study
89	SAN318	SAN118	h+/h- ade6-M210/ade6-M216 ura4-D18/ura4-D18 his3.Δ1/his3.Δ1 leu1::pCAD1-CPR/leu1::pCAD1-UGT2B17/pREP1-CYP2D6	CPR, UGT2B17, CYP2D6	This study
90	SAN319	SAN119	h+/h- ade6-M210/ade6-M216 ura4-D18/ura4-D18 his3.Δ1/his3.Δ1 leu1::pCAD1-CPR/leu1::pCAD1-UGT2B28/pREP1-CYP2D6	CPR, UGT2B28, CYP2D6	This study
**Diploids with CYP4Z1**					
91	SAN500	SAN100	h+/h- ade6-M210/ade6-M216 ura4-D18/ura4-D18 his3.Δ1/his3.Δ1 leu1::pCAD1-CPR/leu1::pCAD1/pREP1-CYP4Z1	CPR, CYP4Z1	This study
92	SAN501	SAN101	h+/h- ade6-M210/ade6-M216 ura4-D18/ura4-D18 his3.Δ1/his3.Δ1 leu1::pCAD1-CPR/leu1::pCAD1-UGT1A1/pREP1-CYP4Z1	CPR, UGT1A1, CYP4Z1	This study
93	SAN502	SAN102	h+/h- ade6-M210/ade6-M216 ura4-D18/ura4-D18 his3.Δ1/his3.Δ1 leu1::pCAD1-CPR/leu1::pCAD1-UGT1A3/pREP1-CYP4Z1	CPR, UGT1A3, CYP4Z1	This study
94	SAN503	SAN103	h+/h- ade6-M210/ade6-M216 ura4-D18/ura4-D18 his3.Δ1/his3.Δ1 leu1::pCAD1-CPR/leu1::pCAD1-UGT1A4/pREP1-CYP4Z1	CPR, UGT1A4, CYP4Z1	This study
95	SAN504	SAN104	h+/h- ade6-M210/ade6-M216 ura4-D18/ura4-D18 his3.Δ1/his3.Δ1 leu1::pCAD1-CPR/leu1::pCAD1-UGT1A5/pREP1-CYP4Z1	CPR, UGT1A5, CYP4Z1	This study
96	SAN505	SAN105	h+/h- ade6-M210/ade6-M216 ura4-D18/ura4-D18 his3.Δ1/his3.Δ1 leu1::pCAD1-CPR/leu1::pCAD1-UGT1A6/pREP1-CYP4Z1	CPR, UGT1A6, CYP4Z1	This study
97	SAN506	SAN106	h+/h- ade6-M210/ade6-M216 ura4-D18/ura4-D18 his3.Δ1/his3.Δ1 leu1::pCAD1-CPR/leu1::pCAD1-UGT1A7/pREP1-CYP4Z1	CPR, UGT1A7, CYP4Z1	This study
98	SAN507	SAN107	h+/h- ade6-M210/ade6-M216 ura4-D18/ura4-D18 his3.Δ1/his3.Δ1 leu1::pCAD1-CPR/leu1::pCAD1-UGT1A8/pREP1-CYP4Z1	CPR, UGT1A8, CYP4Z1	This study
99	SAN508	SAN108	h+/h- ade6-M210/ade6-M216 ura4-D18/ura4-D18 his3.Δ1/his3.Δ1 leu1::pCAD1-CPR/leu1::pCAD1-UGT1A9/pREP1-CYP4Z1	CPR, UGT1A9, CYP4Z1	This study
100	SAN509	SAN109	h+/h- ade6-M210/ade6-M216 ura4-D18/ura4-D18 his3.Δ1/his3.Δ1 leu1::pCAD1-CPR/leu1::pCAD1UGT1A10/pREP1-CYP4Z1	CPR, UGT1A10, CYP4Z1	This study
101	SAN510	SAN110	h+/h- ade6-M210/ade6-M216 ura4-D18/ura4-D18 his3.Δ1/his3.Δ1 leu1::pCAD1-CPR/leu1::pCAD1-UGT2A1/pREP1-CYP4Z1	CPR, UGT2A1, CYP4Z1	This study
102	SAN511	SAN111	h+/h- ade6-M210/ade6-M216 ura4-D18/ura4-D18 his3.Δ1/his3.Δ1 leu1::pCAD1-CPR/leu1::pCAD1-UGT2A2/pREP1-CYP4Z1	CPR, UGT2A2, CYP4Z1	This study

103	SAN512	SAN112	h+/h- ade6-M210/ade6-M216 ura4-D18/ura4-D18 his3.Δ1/his3.Δ1 leu1::pCAD1-CPR/leu1::pCAD1-UGT2A3/pREP1-CYP4Z1	CPR, UGT2A3, CYP4Z1	This study
104	SAN513	SAN113	h+/h- ade6-M210/ade6-M216 ura4-D18/ura4-D18 his3.Δ1/his3.Δ1 leu1::pCAD1-CPR/leu1::pCAD1-UGT2B4/pREP1-CYP4Z1	CPR, UGT2B4, CYP4Z1	This study
105	SAN514	SAN114	h+/h- ade6-M210/ade6-M216 ura4-D18/ura4-D18 his3.Δ1/his3.Δ1 leu1::pCAD1-CPR/leu1::pCAD1-UGT2B7/pREP1-CYP4Z1	CPR, UGT2B7, CYP4Z1	This study
106	SAN515	SAN115	h+/h- ade6-M210/ade6-M216 ura4-D18/ura4-D18 his3.Δ1/his3.Δ1 leu1::pCAD1-CPR/leu1::pCAD1-UGT2B10/pREP1-CYP4Z1	CPR, UGT2B10, CYP4Z1	This study
107	SAN516	SAN116	h+/h- ade6-M210/ade6-M216 ura4-D18/ura4-D18 his3.Δ1/his3.Δ1 leu1::pCAD1-CPR/leu1::pCAD1-UGT2B11/pREP1-CYP4Z1	CPR, UGT2B11, CYP4Z1	This study
108	SAN517	SAN117	h+/h- ade6-M210/ade6-M216 ura4-D18/ura4-D18 his3.Δ1/his3.Δ1 leu1::pCAD1-CPR/leu1::pCAD1-UGT2B15/pREP1-CYP4Z1	CPR, UGT2B15 CYP4Z1	This study
109	SAN518	SAN118	h+/h- ade6-M210/ade6-M216 ura4-D18/ura4-D18 his3.Δ1/his3.Δ1 leu1::pCAD1-CPR/leu1::pCAD1-UGT2B17/pREP1-CYP4Z1	CPR, UGT2B17, CYP4Z1	This study
110	SAN519	SAN119	h+/h- ade6-M210/ade6-M216 ura4-D18/ura4-D18 his3.Δ1/his3.Δ1 leu1::pCAD1-CPR/leu1::pCAD1-UGT2B28/pREP1-CYP4Z1	CPR, UGT2B28, CYP4Z1	This study
